# Neuroimaging Features of Optic Nerve Hemangioblastoma Identified by Conventional and Advanced Magnetic Resonance Techniques: A Case Report and Literature Review

**DOI:** 10.3389/fonc.2021.763696

**Published:** 2021-11-12

**Authors:** Meihan Duan, Lie Yang, Jun Kang, Renzhi Wang, Hui You, Ming Feng

**Affiliations:** ^1^ School of Medicine, Tsinghua University, Beijing, China; ^2^ Department of Neurosurgery, Beijing Tongren Hospital, Capital Medical University, Beijing, China; ^3^ Department of Neurosurgery, Peking Union Medical College Hospital, Chinese Academy of Medical Sciences and Peking Union Medical College, Beijing, China; ^4^ Department of Radiology, Peking Union Medical College Hospital, Chinese Academy of Medical Sciences and Peking Union Medical College, Beijing, China

**Keywords:** neuroimaging, magnetic resonance imaging (MRI), optic nerve hemangioblastoma, diffusion-weighted imaging (DWI), apparent diffusion coefficient (ADC), angiography, edema

## Abstract

Optic nerve hemangioblastoma is a very rare benign tumor with only 39 reported cases by now. It appears to be hyperintense on T2-weighted images with a significant enhancement on contrast scans, which are similar to glioma and meningioma. Due to the lack of specificity in MRI manifestations, optic nerve hemangioblastoma is often misdiagnosed. To provide new insights into differential diagnosis of optic nerve hemangioblastoma, we report for the first time an optic nerve hemangioblastoma case employing advanced magnetic resonance techniques including diffusion-weighted imaging (DWI), apparent diffusion coefficient (ADC) maps, and magnetic resonance angiography (MRA). In addition, we have collected all reported optic nerve hemangioblastoma cases and reviewed their neuroimaging findings by MRI and angiography. Our results show that solid-type tumor is the dominant form of optic nerve hemangioblastoma and extensive edema is widely observed. These findings are surprisingly contrary to manifestations of cerebellar hemangioblastoma. Besides the structural features, quantitative indexes including ADC and relative cerebral blood volume (rCBV) ratio, which are significantly elevated in cerebellar hemangioblastoma, may also shed a light on the preoperative diagnosis of hemangioblastoma of optic nerve. Finally, we discuss the critical neuroimaging features in the differential diagnosis between optic nerve hemangioblastoma from optic pathway glioma and optic nerve sheath meningioma.

## Introduction

Hemangioblastoma is an uncommon benign tumor of central nervous system (CNS), accounting for 2% of all CNS tumors and 7%–12% of all posterior fossa tumors (1, 2). Composed of capillary vessels and stromal cells, it is classified as mesenchymal nonmeningothelial tumors of unknown origin and graded as WHO I. Generally, it occurs in the brain (90%) or spinal cord (10%). Among the intracranial cases, 95% locate in the posterior fossa. Hemangioblastoma at other locations are very rare especially the ones involving optic nerves and chiasm. Common clinical symptoms include headache, cerebellar ataxia, and dyskinesia, which are related to its anatomic locations. Hemangioblastoma can be sporadic (~75%) or von-Hippel Lindau (VHL) syndrome-associated (~25%) (3). VHL syndrome is an autosomal dominant disease characterized by multiple tumors such as hemangioblastoma, renal cell carcinoma, and pheochromocytoma (3). Based on the components, hemangioblastoma can be classified into four types, cystic with mural nodule (54.8%), solid (28.8%), cystic (12.5), and solid with cystic components (3.8%) (4).

Optic nerve hemangioblastoma (ONH) is a very rare subtype with only 39 cases reported. Unlike hemangioblastoma in cerebellum and other locations, most ONH (71%) were found to be associated with VHL syndrome. Visual defects and exophthalmos are the most common symptoms (5). However, these symptoms are unspecific and therefore have limited values in diagnosis. Though MRI with contrast is considered the optimal diagnostic tool by now, ONH is still often misdiagnosed or undiagnosed preoperatively due to its low prevalence and similar MRI patterns to glioma and meningioma. More neuroimaging clues are required to improve preoperative diagnosis.

Here, we employ advanced MR techniques including diffusion weighted imaging (DWI), apparent diffusion coefficient (ADC) maps and MR angiography (MRA) in an ONH case, expecting to offer more imaging information on the preoperative diagnosis. To our knowledge, it is the first reported ONH case characterized by these advanced MR techniques. Also, we have collected all other published ONH cases, summarized their neuroimaging findings, and compared them with other easily confused diseases of optic nerve such as optic pathway glioma and optic nerve sheath meningioma.

## Case Report

A 41-year-old male with a past history of cerebellar hemangioblastoma complained of vision loss in the left eye for 6 years, with reduced vision acuity in the right eye for 3 months. His vision acuity in the left eye started to deteriorate 6 years ago without other discomforts. CT scans at that time showed a mass at cerebellum. He underwent a resection surgery but the left eye still went blind several months later. Postoperative pathological examination confirmed the diagnosis of hemangioblastoma. Two years ago, MRI revealed a newly occurring mass adjacent to the left optic nerve which was asymptomatic. Unfortunately, the follow-up visits showed that it had been progressing gradually without treatment.

**Figure 1:**
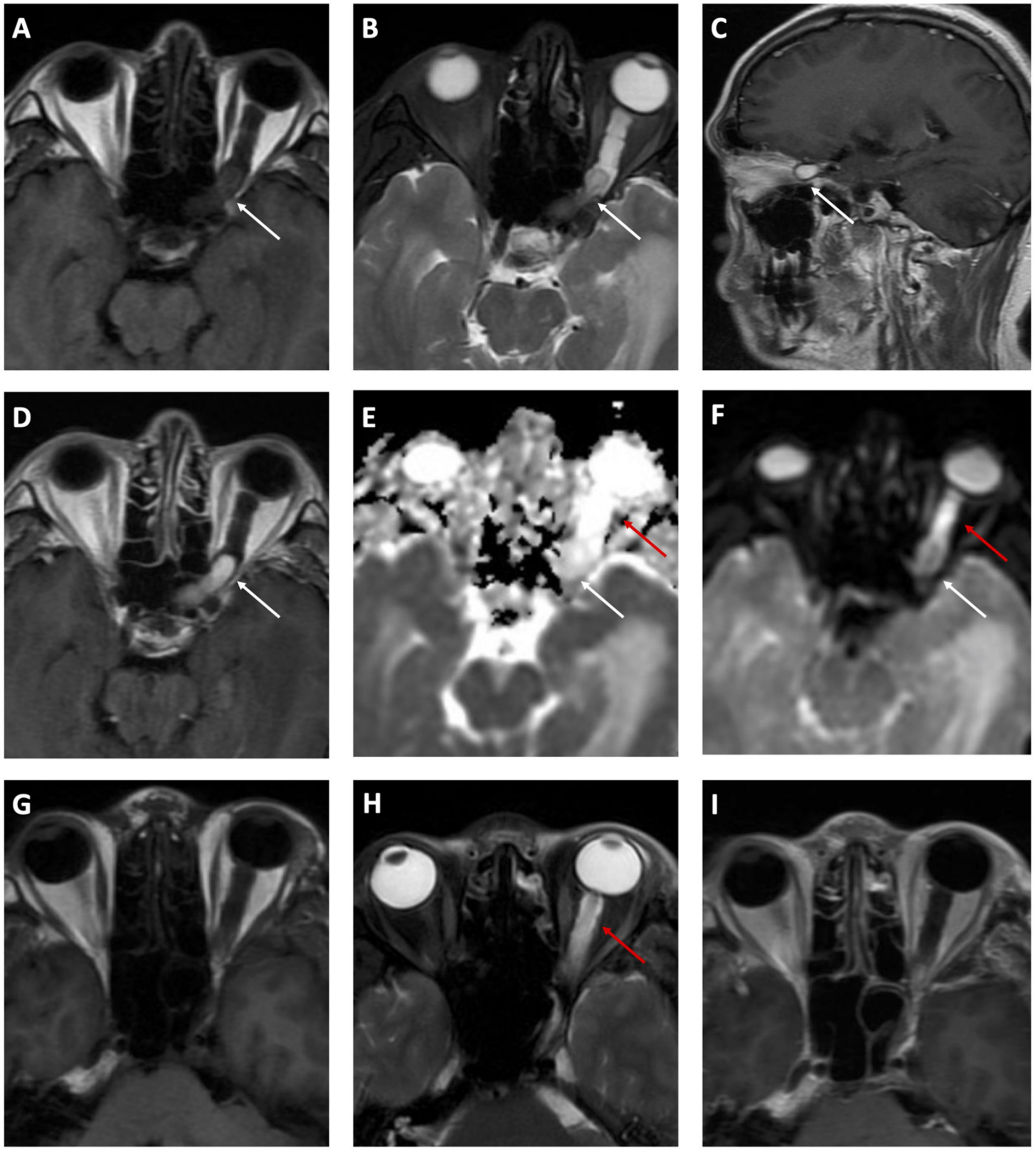
Preoperative **(A–F)** and postoperative **(G–I)** MRI. A hypointense mass (white arrows) from the left orbit apex to the anterior cranial fossa is shown on T1-weighted axial **(A)** MRI scan. It is hyperintense on T2-weighted axial **(B)** and sagittal **(C)** MRI scans, with a significant enhancement on contrast images **(D)**. Obvious hyperintensity is identified on the ADC map **(E)** but not on DWI **(F)**. The mass is absent on both T1WI **(G)** and T2WI **(H)** while edema (red arrows) recedes incompletely. The contrast scan **(I)** shows no enhancement indicating the mass was excised completely.

Three months ago, his right eye had a significant reduction in vision acuity from 20/20 to 20/25 and was referred to our hospital. The visual field test and fundoscopy revealed no abnormality in the right eye. MRI revealed a mass occupying from the left orbit apex to the anterior cranial fossa, with hypointensity on T1-weighted images and hyperintensity on T2-weighted images (**Figures 1A–C**). Apparent and homogenous enhancement of the mass was observed on the gadolinium-enhanced MRI scans (**Figure 1D**), while the surrounding area showed no enhancement. The mass showed evident hyperintensity compared to brain parenchyma on ADC maps (**Figure 1E**) but not on DWI (**Figure 1F**). The surrounding area along the left optic nerve was hyperintense on DWI (**Figure 1G**), and was considered severe peritumoral edema. Also, bilateral optic tracts were thickened on MRI due to swelling. MRA revealed no abnormity (*Supplementary Material*). Taken together, these imaging results highly suggested glioma. Hemangioblastoma was also suspected considering his past history. The latest MRI showed another mass in the left cerebellum, which was hyperintense on T2-weighted imaging (T2WI) and hypointense on T1-weighted imaging (T1WI) and DWI, with significant enhancement on contrast scans. These MRI findings and the location of posterior fossa strongly supported the possibility of hemangioblastoma. Combining these imaging clues and his past history, the recurrence of hemangioblastoma at both cerebellum and optic nerve was highly suspected.

The patient then received a left frontotemporal-approach craniotomy to resect the mass. At surgery, a reddish tumor adherent to the left optic nerve was observed. The attached tortuous vessels originated from ophthalmic artery indicated its rich blood supply. The tumor was excised and measured 1.5 × 0.8 × 0.8 cm. Postoperative MRI (**Figures 1H, I** **)** showed a complete excision of the mass. The edema has receded postsurgery but not completely. Changes in vision or visual fields have not been observed during the 2-week hospital admission after surgery.

**Figure 2:**
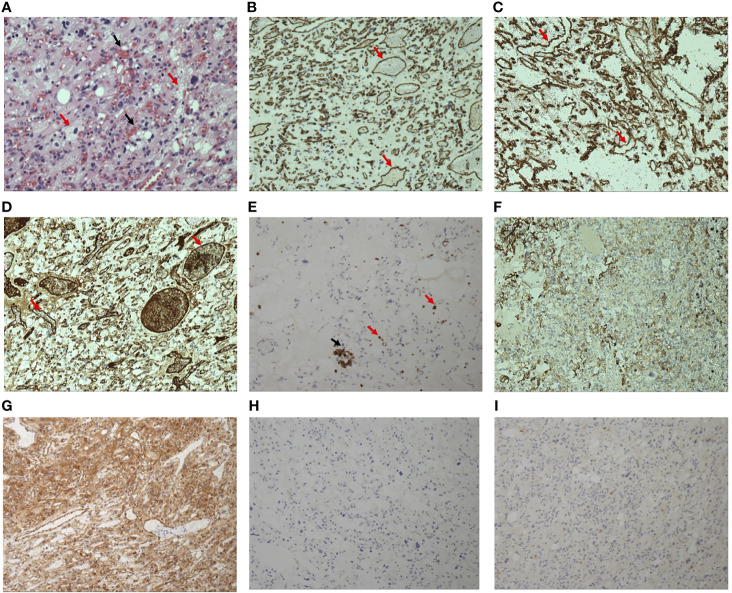
Hematoxylin-eosin staining of the excised mass (×20) **(A)** shows numerous capillary vessels containing red blood cells (black arrows) distributed in the vacuolated stromal cells (red arrows). Immunohistochemistry staining of the mass shows that the endothelial cells of capillary vessels (red arrows) were positive (brown=positive) for CD31 **(B)**, CD34 **(C)**, and F8-R **(D)**. Some interspersed monocytes (red arrows) and macrophages (black arrow) were positive for CD68 **(E)**. Vacuolated stromal cells are positive for GFAP **(F)** and NSE **(G)**. CgA **(H)** and Syn **(I)** is negative among the whole sample tissue.

Macroscopically, the mass was solid, soft, and had a taupe surface. Histopathological results revealed that the tumor consisted of vacuolated stromal cells and numerous capillary vessels. The stromal cells are NSE(+) and GFAP(+), and the vessels are CD34(+), CD31(+), and F8-R(+). Interspersed CD68(+) monocytes and macrophages were also observed. CgA and Syn were both negative among the whole tissue (**Figure 2**). Therefore, the diagnosis of hemangioblastoma was confirmed by the typical pathological characteristics. Considering the frequent recurrence of hemangioblastoma in this patient, VHL syndrome was probably associated with his clinical manifestations. Unfortunately, the patient refused genetic testing.

## Neuroimaging in Optic Nerve Hemangioblastoma

We collected all 39 published ONH cases and reviewed their neuroimaging results.

### The MRI Findings of Optic Nerve Hemangioblastoma

MRI findings of ONH are shown in **Table 1**. Although most research groups performed MRI on ONH patients, few of them described detailed results. Since Ginzburg et al. (14) firstly applied MRI in an ONH patient, almost all subsequent cases have reported prominent enhancement on gadolinium-enhancing scans, 14 homogenous and nine heterogenous. Nine cases were reported to show significant hyperintense mass on T2WI. In the eight cases reporting T1WI, five cases presented isointense mass compared with brain parenchyma, while the other three are hypointense. Thus, a well-defined boundary on T2WI and enhanced scans was commonly observed.

**Table 1:** MRI findings of 40 published optic nerve hemangioblastoma cases.

Year	First author	T1WI	T2WI	Gd-enhanced	Cystic/solid	Diameter (cm)
1942	Schneider (6)				Solid	3.5
1974	Stefani and Rothemund (7)				Solid	NA
1981	Lauten (8)				Solid	1.5
1981	Eckstein (9)				Solid	2.3
1982	In (10)				Solid	2.9
1984	Tanaka (11)				Solid	2.5
1988	Nerad (12)				Solid	NA
1989	Hotta (13)				Solid	NA
1992	Ginzburg (14)	NA	NA	Homogenous	Solid	NA
1994	Rubio (15)	NA	NA	Homogenous	Solid	3
1995	Balcer (16)	NA	NA	Homogenous	Solid	2.2
1995	Kerr (17)	NA	NA	Enhanced	Solid	1.7
1997	Raila (18)	Iso-	Hyper-	Homogenous	Solid	1
1994	Miyagami (19)	NA	NA	NA	Solid	NA
2000	Kouri (20)	NA	NA	Homogenous	Cystic/solid	1.4
2004	Kato (21)	NA	NA	NA	Solid	NA
2006	Fons Martinez (22)	NA	Hyper-	Homogenous	Solid	NA
2007	Higashida (23)	Iso-	Hyper-	Homogenous	Solid	NA
2008	Barrett (24)	NA	NA	Homogenous	Solid	4.5
2008	Meyerle (25)	Hypo-	Hyper-	Heterogenous	Internal cyst	2.1
		NA	NA	Homogenous	Solid	1.4
		NA	NA	Enhanced	Cystic/solid	1
		NA	NA	Enhanced	Solid	1
		NA	NA	NA	Solid	1.6
		NA	NA	NA	Internal cyst	2.5
		NA	NA	NA	Internal cyst	1.6
2008	Baggenstos (26)	NA	NA	Homogenous	Solid	NA
2009	Prabhu (27)	Iso-	Hyper-	Heterogenous	Solid	3.5
2010	Shima (28)	NA	NA	Heterogenous	Solid	2.6
2012	Zywicke (29)	NA	NA	Heterogenous	Cystic/solid	1
2014	Staub (30)	NA	NA	Heterogenous	Solid	1.2
2014	Fard (31)	NA	NA	Homogenous	Solid	NA
2016	Turel (32)	Hypo-	Hyper-	Heterogenous	Cystic/solid	1.8
2018	McGrath (33)	NA	Hyper-	Heterogenous	Cystic/solid	0.8
2018	Kanno (34)	NA	NA	Homogenous	Solid	NA
2019	Darbari (35)	Iso-	Hyper-	Homogenous	Cystic/solid	NA
2020	Boratto (36)	NA	NA	Heterogenous	Solid	0.8
2020	Xu (37)	Iso-	Hyper-	Heterogenous	Cystic/solid	NA
2021	Present	Hypo-	Hyper-	Homogenous	Solid	1.5

NA, not available.

The typical form of cerebellar hemangioblastoma is a large cyst with mural nodules, accounting for 54.8%, while the solid form composes about 28.8% (4). However, the situation differs in ONH. For all 40 ONH cases, 75% are solid and only 25% are found to have cystic components. The maximum sizes between the two groups are not significantly different.

In addition, moderate to severe peritumoral edema is a widely accepted and exclusive feature of ONH, but not observed in cerebellar hemangioblastoma. It is known that the presence of edema is associated with tumor malignancy (38). ONH as a benign tumor however, comes with frequent and extensive peritumoral edema, which possibly results from the immature neovascular tissues and its high permeability. Given the fact that most symptoms of brain tumors result from their compression, the extensive edema involving a large region may cause severe symptoms beyond the reach of the mass itself. Several groups reported that edema could expand broadly along the low-pressure space, invading optic chiasma and even contralateral optic nerve, leading to the impairment of bilateral vision (26, 30, 32, 33). Even worse, Xu et al. has reported a case with bilateral frontal lobe edema (37). Regardless of the prognosis of the affected side, timely excision is always necessary for avoiding further expansion of edema and retaining the visual function of unaffected side. In addition, several groups discovered that the edema receded rapidly after operations (26, 32), suggesting that the surrounding compressed areas are recoverable.

The dominant solid form and frequent broad edema are specific to ONH instead of cerebellar hemangioblastoma, which can be potentially explained by its location. The pressure is relatively low along the optic nerves and optic chiasma so that the transudate of ONH can be drained smoothly. In contrast, the cerebellar tissue is dense and compact, forcing the transudate to compress surrounding areas. Therefore, the cerebellar lesion would eventually form a cyst with nodules. Though further investigation is needed to prove the mechanism, these features can nevertheless remind us more of ONH when facing the differential diagnosis of optic nerves tumors.

The flow void effect is another specific feature of ONH. Flow void effect is characterized by the signal loss on MRI occurring with flowing substances like blood and urine. It is caused by the displacement of the moving/flowing substances in the time of flight between the photon excitation and scanning. Due to the flow void effect, flowing blood can appear as grey to black on T2WI, forming voids in the bright area of relatively immobile surrounding tissues. Meyerle et al. has investigated nine ONH cases, and five out of nine showed flow voids. Flow void-positive masses possess larger maximum diameters than the others, though the sample size was too small to draw a conclusion (25). The presence of flow voids highly suggests hemangioblastoma because of its exclusively rich vascular components. While for other tumors, flow voids occur only when the blood supply of tumors is extremely rich and the tumor is accordingly very large in size.

Besides the conventional sequences, advanced MRI techniques such as DWI and ADC have been used to differentiate brain tumors. Several studies have investigated the values of DWI and ADC in the differential diagnosis of hemangioblastoma and other common tumors in the posterior fossa, yet optic nerve tumors have not been focused on. Most studies concluded that the ADC values of hemangioblastoma were significantly higher than those of metastatic tumors (39–42). Hemangioblastoma is also found to have higher ADC values than most primary tumors including meningioma and medulloblastoma, except pilocytic astrocytoma (PA) according to Payabvash and colleagues (40). As the only ONH case with DWI and ADC results, the case reported in this paper seems to be apparently hyperintense on ADC maps, similar to the findings in cerebellar hemangioblastoma. Collectively, these sequences combined with quantification can potentially be used as diagnostic tools for recognizing ONH, especially for differentiating ONH from metastatic tumors and meningioma.

Perfusion-weighted imaging (PWI) is another superior method derived from MRI. Hakyemez et al. (43) and She et al. (42) found that hemangioblastoma had extremely high relative cerebral blood volume (rCBV) ratio compared with other common tumors in the posterior fossa including gliomas, meningiomas, lymphomas, and metastases. Though the modality has not been used in ONH, its hypervascularity nature may prompt a high rCBV in ONH. As an objective index, PWI could be of great value in the differential diagnosis of ONH and optic pathway glioma.

### The Results of Angiography in Optic Nerve Hemangioblastoma

In the early time when MRI has not been widely used, angiogram was a useful tool for the diagnosis of brain lesions and identifying feeding arteries and draining veins, which were crucial for successful operations. Seven ONH cases (9, 10, 13, 18, 23, 24, 30) reported the results of angiogram, and a hypervascular mass with heavy staining was often observed. This property helped identify ONH from hypovascular mass like schwannomas, neuritis, and some types of glioma. Angiogram revealed that blood supply of ONH mainly comes from ophthalmic artery, while some cases also reported main feeding arteries including meningohypophyseal trunk, superficial temporal arteries, and internal maxillary arteries. With the advance of MRI technology, the use of angiogram gradually vanishes. However, as the only ONH case with MRA results, we did not find any abnormity in MRA. It was probably due to the lower resolution of MRA compared with angiogram. Some groups have investigated the MRA findings of hemangioblastoma in the posterior fossa and spinal cord, which may also informative for ONH (44–46). Liu et al. (46) utilized the technique of time-resolved MRA (TR MRA) in intracranial vascular lesions. They found that cerebellar hemangioblastoma showed no enhancement in the early arterial phase, and a tumor blush was observed in the capillary and venous phases. Meanwhile, meningioma presented strong enhancement in both early and late arterial phase and venous phase (46). It could be inferred that TR MRA manifestation of ONH would present similarly and can help differentiate meningioma in certain cases.

### Differential Diagnosis of Optic Nerve Hemangioblastoma Based on the Neuroimaging Features

Based on the location at optic pathway and the MRI performances, optic pathway glioma (OPG) and optic nerve sheath meningioma (ONSM) are two commonly considered differential diagnoses of ONH. Here, we summarized the key points in the differential diagnosis based on abovementioned neuroimaging features (**Table 2**).

**Table 2:** Differential diagnosis of ONH, OPG, and ONSM based on typical neuroimaging features.

Typical appearances of optic nerve lesions on conventional MRI scans					
	ONH	OPG	ONSM
Location	IO (73%) (5); IC (24%)	Anterior (40.4%) (47): IO only (78.6%); IO+IC (21.4%); posterior (59.6%)	IO only (71.6%); IO+IC (28.4%) (48)
Pattern	Solid mass (75%); solid and cystic mass (25%)	Fusiform enlargement or uniform thickening of optic nerve (47); solid and cystic components (49)	Tubular (62.2%) or globular (23.0%) enlargement of optic nerve (48); rare cystic changes
T1WI	Isointense or hypointense	Isointense (49)	Isointense (50)
T2WI	Hyperintense	Hyperintense (49)	Hyperintense (50)
Gadolinium contrast	Solid: enhancing; cysts: nonenhancing	Solid: enhancing and nonenhancing; cystic: nonenhancing (49)	Enhancing (50)
Specific sign	Flow void (5)		Tram track (50)
Peritumoral edema	Common; extensive	Rare	Rare		
Referential results of cerebellar counterpart on advanced MRI sequences					
	Hemangioblastoma	Low-grade glioma	Meningioma
ADC (39, 40)	Medium	High (PA)	Low
rCBV (43)	High (11.36)	Low (1.69)	Medium (8.02)

IO, intraorbital; IC, intracranial.

The OPG is a kind of low-grade glioma which occurs majorly in children. Histologically, most OPGs are classified as PA. PA typically appears as fusiform enlargement or uniform thickening of optic nerve (47). It has a very similar intensity pattern to ONH on MRI (i.e., isointense on T1WI and hyperintense on T2WI). Its enhancement is often heterogeneous due to the mixture of cystic, enhancing and nonenhancing solid components (49). Though there is no published case comparing PA and hemangioblastoma in optic nerve, evidences show that cerebellar PA has higher ADC values than hemangioblastoma (40).

There are three major points that may contribute to the differentiation of OPG and ONH: (1) flow voids as the most specific marker of ONH are rarely seen in OPG; (2) prominent peritumoral edema is also suggestive of ONH. The OPG, like most low-grade tumors, induces no or little peritumoral edema (51); (3) the presence of nonenhancing solid and cystic components is more commonly seen in OPG. With the advancement of PWI technique, the significant differences of rCBV between hemangioblastoma and PA may greatly help in diagnosing the optic nerve lesions. In a comparative study of PA and hemangioblastoma in cerebellum, the rCBV value of PA and high-grade glioma were measured 1.69 and 5.76, respectively, while that of hemangioblastoma was significantly higher at 11.36 (43). Therefore, PWI is fairly promising in the differentiation of brain tumors with similar performances on conventional MRI sequences. Besides MRI performances, clinical features such as young age and stable biological behaviors are more likely to support the diagnosis of OPG.

ONSM is a rare benign tumor originating from meningoepithelial cap cells of arachnoid villi. More than 90% ONSM are intraorbital. While it has a similar intensity pattern to ONH on MRI, ONSM usually appears as tubular or globular enlargement of optic nerve (48). Cystic changes are rarely seen. Also, ONSM has a specific MRI sign named “tram track,” which is characterized by the homogenous and marked enhancement of meningioma flanking the hypointense optic nerve on contrast MRI, mimicking the appearance of a tram track. The obvious calcification on noncontrast CT scans also suggests ONSM instead of ONH (50). Results of advanced MRI techniques may further confirm the diagnosis of meningioma when other findings are not typical, such as relatively low ADC and rCBV values.

## Discussion and Conclusion

Optic nerve hemangioblastoma is extremely rare. Including this case, 40 cases have been reported since 1940. Despite the fact that MRI is the major diagnostic tool of ONH, few of these case reports provided detailed descriptions of MRI results for reference in clinical practice. On the other hand, ONH has similar MRI performances to OPG and ONSM, making it highly challenging to accurately diagnose ONH. Therefore, more experience on MRI imaging is needed to improve the diagnosis. In this study, we reported a 41-year-old male with recurrent hemangioblastoma in both cerebellum and optic nerve. The manifestations on conventional MRI were described in detail. Moreover, advanced MR techniques were employed for the first time to explore for more diagnostic tools for ONH. The results revealed that the mass was evidently hyperintense on ADC maps but showed no abnormity on MRA, which was out of expectation.

We also reviewed all present case reports to summarize the MRI and angiography results of ONH and discussed the differential points between ONH and easily confused diseases. Similar to cerebellar hemangioblastoma, most ONH cases possess iso- or hypointensity on T1WI and hyperintensity on T2WI. The prominent enhancement and well-defined boundaries are prevalent. In addition, structural features including flow voids and extensive peritumoral edema are also widely accepted as suggestive evidences of ONH. The flow void effect has high specificity but is not sensitive enough. Larger masses seem to be more likely to cause the effect. Extensive peritumoral edema is a unique property of ONH absent in cerebellar hemangioblastoma. Optic pathway is often involved due to its low resistance environment and contralateral vision can be impaired consequently, indicating the necessity of timely and complete resection. It is also found that the solid-form hemangioblastoma accounts for the vast majority of ONH, instead of cystic form which typically presents in cerebellar hemangioblastoma. This possibly resulted from drainage of transudate through the extensive edema. Therefore, the absence of typical large cysts with mural nodules should not be recognized as a clue for excluding ONH during diagnostic process.

ONH is difficult to be distinguished from meningioma and glioma with simply conventional imaging approaches, especially when typical clinical features mentioned above are not observed. Though few studies and reports focus on applying advanced MRI techniques on ONH, experience from cerebellar hemangioblastoma can also be informative. It has been reported that ADC values in hemangioblastoma are distinctively higher when compared with most other posterior fossa tumors such as meningioma and metastases, indicating its potential diagnostic value in ONH. We here applied ADC on the evaluation of ONH for the first time and observed a hyperintense mass, similar to the manifestations of cerebellar hemangioblastoma.

In addition, high rCBV ratio on PWI can be another exclusive marker of hemangioblastoma. Cerebellar hemangioblastoma shows a remarkably higher rCBV ratio than metastases, meningioma, and both low-grade and high-grade gliomas, and thus can be easily distinguished. However, PWI results in ONH have not been reported yet.

Angiography used to be crucial for the diagnosis of ONH before MRI was advanced. Almost all cases performing angiogram revealed hypervascular masses originating from ophthalmic arteries. In contrast, MRA has not been frequently used in either optic nerve or cerebellar hemangioblastoma.

In conclusion, various MR modalities including DWI, ADC, and PWI provide new insights to the imaging manifestations of ONH. For diagnosed VHL patients, an optic nerve lesion is highly suspected to be hemangioblastoma. However, for patients who do not meet the clinical diagnosis criteria of VHL, any possibility of the lesion should be carefully evaluated. In such cases, preoperative diagnosis by advanced MRI techniques can be decisive for their following examinations, treatment, and prognosis. Though more ONH cases employing these advanced techniques are needed to systematically review their diagnostic performances, these tools are valuable auxiliary approaches that can prompt the differentiation of ONH in these situations.

## Data Availability Statement

The original contributions presented in the study are included in the article/**Supplementary Material**. Further inquiries can be directed to the corresponding authors.

## Ethics Statement

Written informed consent was obtained from the individual(s) for the publication of any potentially identifiable images or data included in this article.

## Author Contributions

MD composed the manuscript and reviewed literature. LY provided figures and reviewed literature. JK and RW contributed to the organization and revision of the manuscript. HY reviewed the MRI images in case reports, analyzed the results, and edited the manuscript. MF selected and managed the patient case and edited the manuscript. All authors contributed to the article and approved the submitted version.

## Funding

This work was supported by Chinese Academy of Medical Sciences / Peking Union Medical College Postgraduate Teaching Innovation Fund (No. 10023201900107).

## Conflict of Interest

The authors declare that the research was conducted in the absence of any commercial or financial relationships that could be construed as a potential conflict of interest.

## Publisher’s Note

All claims expressed in this article are solely those of the authors and do not necessarily represent those of their affiliated organizations, or those of the publisher, the editors and the reviewers. Any product that may be evaluated in this article, or claim that may be made by its manufacturer, is not guaranteed or endorsed by the publisher.

## Abbreviations

CNS, central nervous system; MRI, magnetic resonance imaging; VHL syndrome, von Hippel-Lindau syndrome; ONH, optic nerve hemangioblastoma; DWI, diffusion-weighted imaging; ADC, apparent diffusion coefficient; MRA, magnetic resonance angiography; T1WI, T1-weighted imaging; T2WI, T2-weighted imaging; TR MRA, time-resolved magnetic resonance, PWI, perfusion-weighted imaging; rCBV, relative cerebral blood volume; OPG, optic pathway glioma; PA, pilocytic astrocytoma; ONSM, optic nerve sheath meningioma.
