# Supplementary material for: Neuroimaging Features of Optic Nerve Hemangioblastoma Identified by Conventional and Advanced Magnetic Resonance Techniques: A Case Report and Literature Review
Source: Front Oncol. 2021 Nov 12;11:763696. doi: 10.3389/fonc.2021.763696 (PMC8632699; doi:10.3389/fonc.2021.763696)
Supplement: Supplementary file 1 [file DataSheet_1.docx]

Supplementary Material

# Supplementary Figure


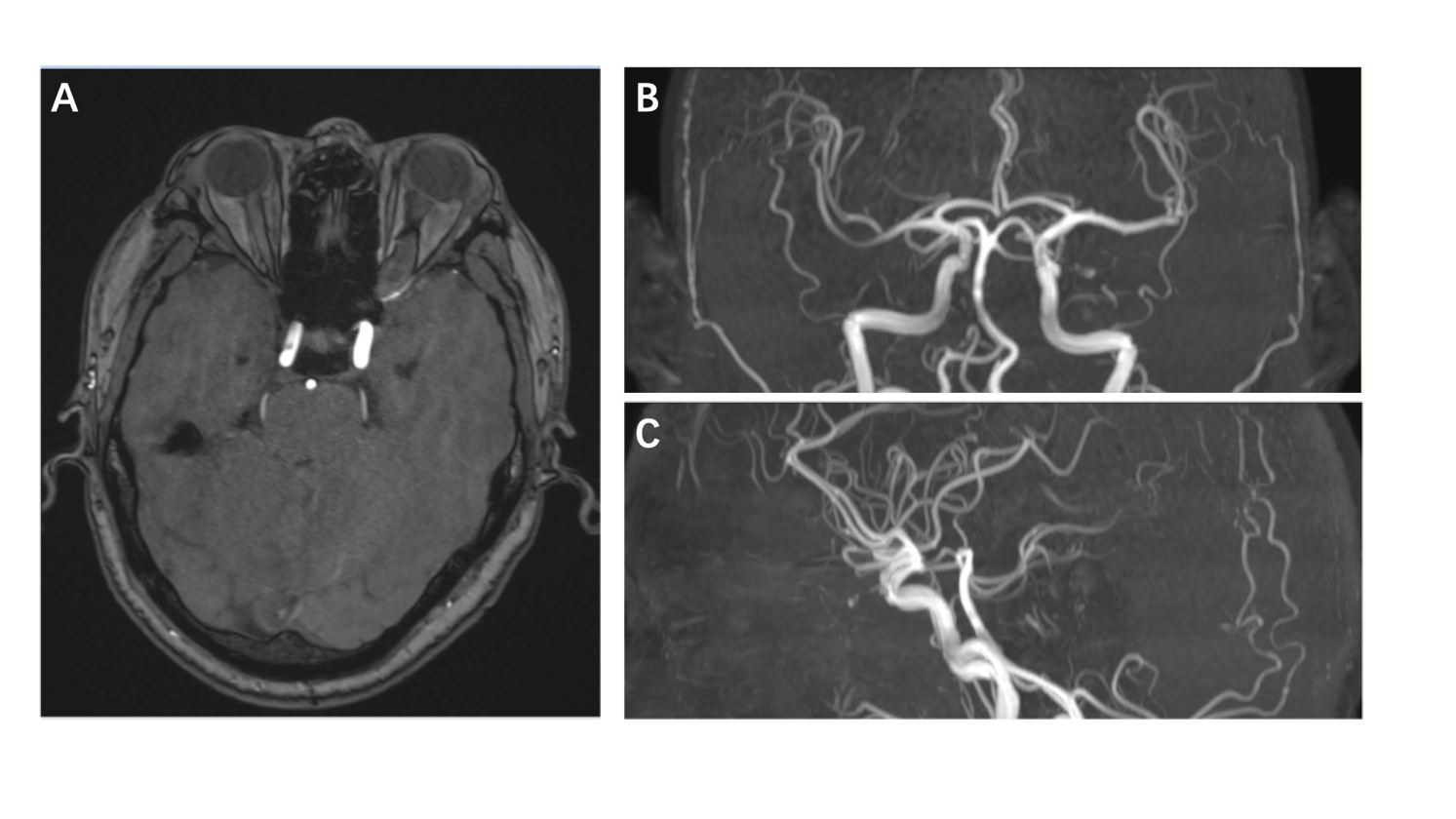


**Supplementary Figure 1.** The preoperative axial MRA scan (**A**) and the reconstructed MRA images (**B, C**) shows no abnormity.
